# A three-dimensional clinical teaching model integrating CBL and PBL for ophthalmology clerkship in traditional Chinese and Western medicine

**DOI:** 10.3389/fmed.2025.1743381

**Published:** 2025-12-18

**Authors:** Minghui Zhao, Juan Li, Jiali Liu, Yanyun Jiang, Rui Liu, Wei Zhou, Shaolin Du, Lixia Liu, Ligang Jiang

**Affiliations:** 1 Department of Ophthalmology, Shanghai Municipal Hospital of Traditional Chinese Medicine, Shanghai University of Traditional Chinese Medicine, Shanghai, China; 2 Tungwah Eye Medical Center, Dongguan Tungwah Hospital, Dongguan, China; 3 Department of Ophthalmology, Quzhou Affiliated Hospital of Wenzhou Medical University, Quzhou People’s Hospital, Quzhou, China

**Keywords:** case based learning, integrated traditional Chinese and Western medicine, ophthalmology education, problem-based learning, three-dimensional integrated teaching model

## Abstract

**Background:**

Integrated traditional Chinese and Western medicine has shown clear advantages in the management of ophthalmic diseases. However, misalignment between traditional Chinese medicine syndrome differentiation and Western pathological classification, overemphasis on Western diagnostic and therapeutic procedures, and insufficient training in traditional Chinese medicine techniques continue to constrain teaching quality in ophthalmology.

**Methods:**

This single-center prospective interventional study implemented a three-dimensional integrated traditional Chinese and Western medicine teaching model that combined case-based learning and problem-based learning, and compared teaching outcomes in 156 medical students before and after the intervention.

**Results:**

The new teaching model received a mean satisfaction score of 4.54 ± 0.33 on a five-point Likert scale. Compared with baseline, students showed significantly higher classroom participation (92.0 ± 4.5% compared with 64.0 ± 8.5%, *p* < 0.001), comprehensive examination scores (92.0 ± 4.0 compared with 77.0 ± 5.5, *p* < 0.001), and overall autonomous learning ability scores (4.54 ± 0.33 compared with 3.20 ± 0.45, *p* < 0.001). Additional improvements were observed in independent literature review frequency (3.5 ± 0.4 compared with 1.2 ± 0.3 times per week, *p* < 0.001), acupuncture point location accuracy (95.0% compared with 84.0%, *p* < 0.001), and Western medicine examination scores (95.5 ± 3.1 compared with 80.5 ± 4.2, *p* < 0.001).

**Conclusion:**

The three-dimensional integrated traditional Chinese and Western medicine teaching model effectively enhanced ophthalmology teaching quality and helped cultivate medical students with integrated traditional Chinese and Western medicine competencies, as reflected by improved student engagement, autonomous learning, and clinical skill mastery.

## 1 Introduction

Ophthalmic diseases are characterized by complex etiology and precise diagnosis and treatment processes (1–4). The integrated traditional Chinese and Western medicine (ITCWM) approach has obvious advantages in ophthalmic treatment (5, 6). Traditional Chinese ophthalmology emphasizes the holistic view and the correlation with internal organs, and has unique advantages in the management, prevention, and postoperative rehabilitation of chronic eye diseases (7, 8). Western medicine, on the other hand, relies on advanced equipment and surgical techniques, and has outstanding advantages in the diagnosis of acute eye diseases and the treatment of ocular trauma. The ITCWM treatment model combines the holistic regulation of traditional Chinese medicine with the precise intervention of Western medicine, providing a new treatment path for some intractable ophthalmic diseases (9, 10).

However, there are currently some issues in ophthalmology clinical teaching. Firstly, in the teaching process, traditional Chinese medicine (TCM) syndrome differentiation and Western medicine pathology are often taught in isolation, lacking effective connection and integration (11). This makes it difficult for students to establish an organic connection between the two and form a comprehensive clinical thinking (12). Secondly, practical teaching processes often focus on Western medicine examinations and surgical techniques, neglecting the training of TCM characteristic operations such as acupuncture and tuina. This imbalance not only results in incomplete diagnostic and treatment skills among students but also poses a potential threat to the inheritance and development of TCM characteristic therapies (13). Lastly, students lack sufficient understanding of the concept of integrated traditional Chinese and Western medicine. They can recite the basic theories of both TCM and Western medicine separately, but when faced with actual clinical cases, they struggle to effectively combine TCM’s holistic view with Western medicine’s precise diagnosis and are unable to develop a diagnostic and treatment plan that truly reflects the advantages of integrated traditional Chinese and Western medicine. The reasons behind this phenomenon are that the teaching content fails to deeply and systematically explain the integration points of the two, lacks sufficient high-quality clinical cases as support, and teachers also have deficiencies in guiding students to establish an integrated thinking (11, 14).

This study aims to systematically address the aforementioned challenges in integrated traditional Chinese and Western medicine teaching by establishing a new teaching model, providing support for cultivating compound ophthalmology talents with integrated traditional Chinese and Western medicine capabilities.

## 2 Materials and methods

### 2.1 Study design and setting

This single-center prospective pre–post interventional study was conducted in the Department of Ophthalmology of Shanghai Municipal Hospital of Traditional Chinese Medicine, Shanghai University of Traditional Chinese Medicine. The aim was to evaluate the effectiveness of a new three-dimensional integrated teaching model. Multiple teaching-related indicators were quantitatively measured and compared in the same cohort of students before and after exposure to the new teaching model. The study protocol was approved by the Ethics Committee of Shanghai Municipal Hospital of Traditional Chinese Medicine, Shanghai University of Traditional Chinese Medicine (approval number: 2024SHL-YKYYS-71). Participation was voluntary, and all students provided written informed consent before enrolment. The study was conducted in accordance with the ethical principles of the Declaration of Helsinki.

### 2.2 Participants and sample size

#### 2.2.1 Participants

The study selected students majoring in integrated traditional Chinese and Western medicine from clinical college of Shanghai Municipal Hospital of Traditional Chinese Medicine, Shanghai University of Traditional Chinese Medicine from January 2024 to June 2025. All participants have completed the basic courses of Western and Traditional Chinese Medicine theory.

Inclusion criteria: (1) full-time medical students undertaking a clinical rotation in ophthalmology in our department during the study period; (2) provision of written informed consent and voluntary participation; and (3) no previous clinical rotation experience in ophthalmology.

Exclusion criteria: (1) absence from clinical teaching activities for more than 10% of the rotation period due to vacation or other reasons; or (2) failure to cooperate with data collection.

#### 2.2.2 Sample size

With a significance level *α* = 0.05 and power 1–β = 0.80, and assuming a 10-point improvement in the mean comprehensive examination score as the primary effect size, the required sample size for a paired pre–post comparison was estimated to be 120 students. Allowing for an anticipated dropout rate of 20%, we planned to recruit 204 students. In practice, 156 students returned valid questionnaires, yielding a response rate of 76.5%. All 156 students completed both the educational intervention and the post-intervention assessment and were included in the final analysis.

### 2.3 The intervention

#### 2.3.1 Constructing a three-dimensional teaching model

**Figure 1:**
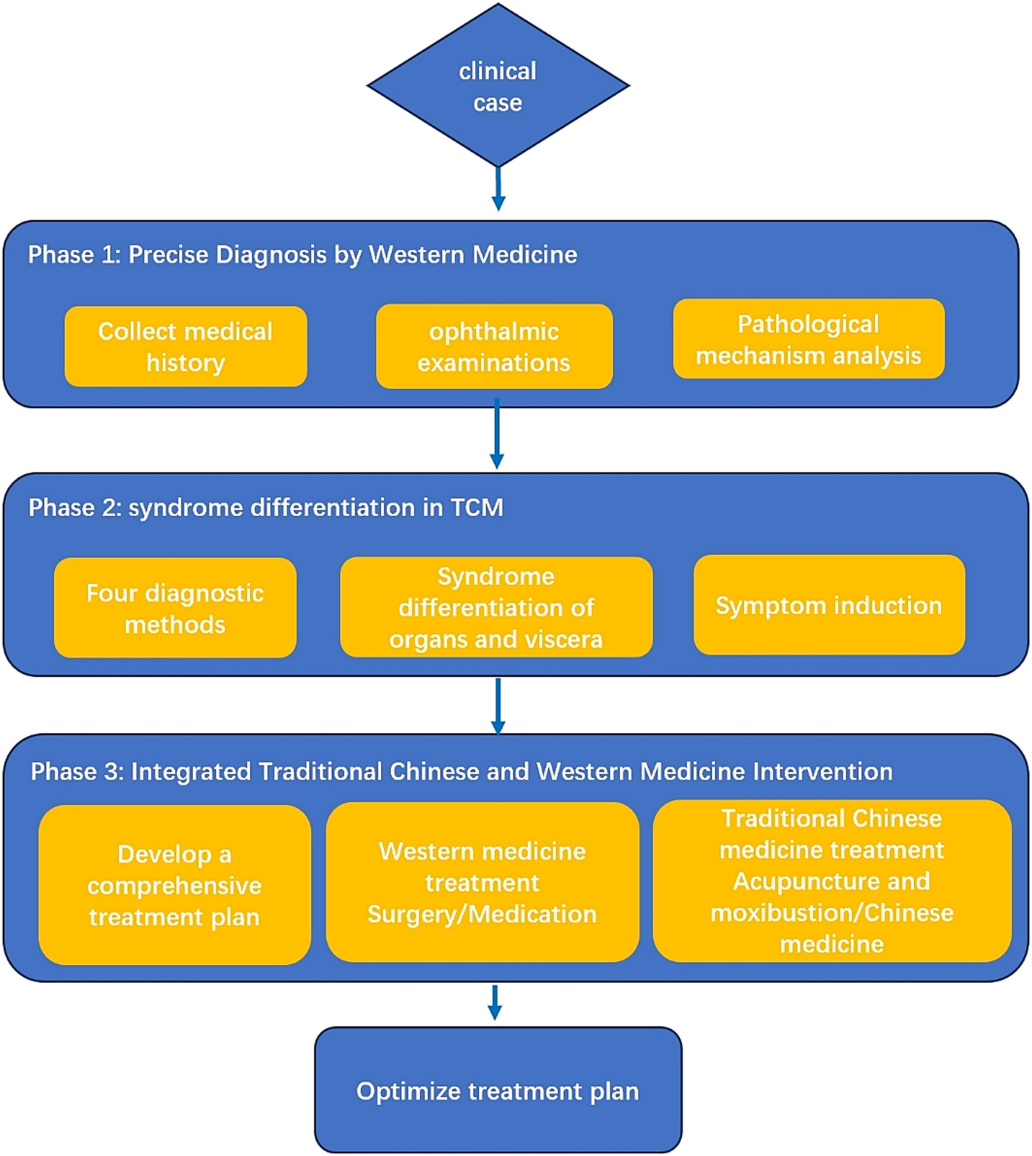
Schematic diagram of three-dimensional integrated traditional Chinese and Western medicine ophthalmology teaching mode.

We designed and implemented a three-dimensional teaching model characterized by “precise Western medicine diagnosis-macroscopic traditional Chinese medicine syndrome differentiation-integrated traditional Chinese and Western medicine intervention.” The total teaching duration for each student was 32 teaching hours. For each clinical case, students were first required to establish a Western medicine diagnosis based on modern ophthalmic theory and examination findings, including visual acuity testing, intraocular pressure measurement, slit-lamp biomicroscopy, and fundus photography. They then applied the four diagnostic methods of traditional Chinese medicine (inspection, listening and smelling, inquiry, and palpation/pulse-taking), combined with the theory of the zang-fu organs, to determine the corresponding traditional Chinese medicine syndrome pattern. Finally, students developed an integrated treatment plan that combined traditional Chinese and Western medicine. For example, in glaucoma cases, the comprehensive plan could include acupuncture and moxibustion at specific acupoints or individualized Chinese herbal formulas tailored to the syndrome pattern, together with intraocular pressure-lowering medications, to protect optic nerve function through synergistic interventions (Figure 1).

#### 2.3.2 Diversified teaching methods

**Figure 2:**
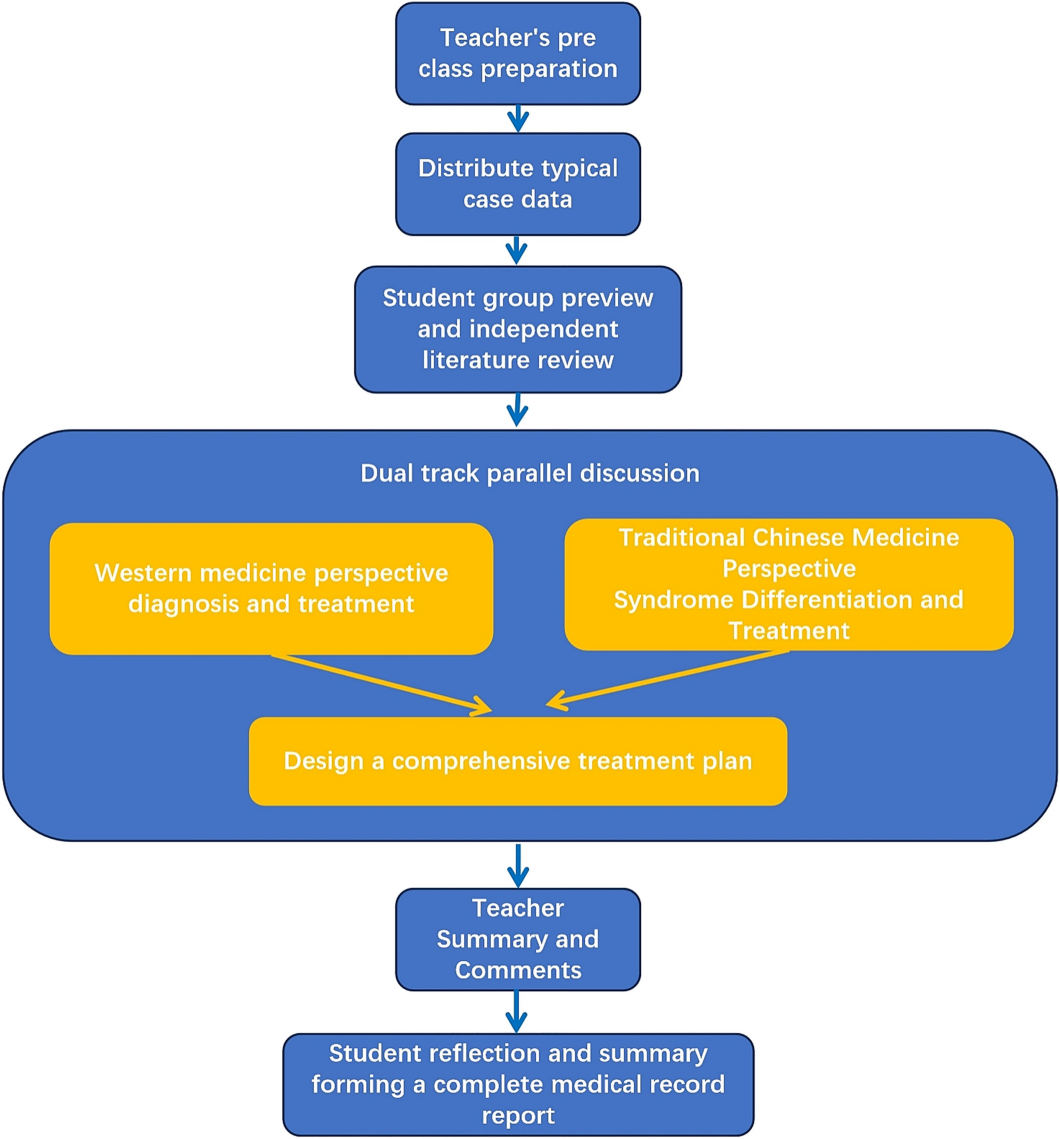
Teaching flowchart combining CBL and PBL.

Two active learning strategies, case-based learning (CBL) and problem-based learning(PBL), were integrated into the teaching model to promote inquiry-driven learning. In the CBL sessions, teachers selected typical ophthalmic cases, such as glaucoma, diabetic retinopathy, or dry eye disease. Case materials were distributed to student groups in advance. During the sessions, students analyzed each case from both Western medicine and traditional Chinese medicine perspectives and designed an integrated treatment plan, after which the teachers facilitated a summary and feedback. In the problem-based learning sessions, teachers posed guiding questions aligned with the learning objectives, such as “Explain the pathogenesis of dry eye disease from the perspective of integrated traditional Chinese and Western medicine, “or “How should acute conjunctivitis be managed using an integrated traditional Chinese and Western medicine approach? “Students independently searched the literature and presented their findings in group presentations to cultivate research thinking and problem-solving skills (Figure 2).

#### 2.3.3 Teaching environment and team building

We have established an ophthalmology training center that integrates traditional Chinese and Western medicine. It is equipped with Western medical devices such as OCT and fundus cameras, and also features a dedicated area for traditional Chinese medicine treatments, equipped with acupuncture needles, Chinese herbal iontophoresis devices, and fumigation therapy equipment. Students can receive systematic and standardized training here, ranging from basic examinations to complex treatments.

Meanwhile, we have assembled a cross-disciplinary teaching team consisting of four teachers, including two Western medicine ophthalmologists and two traditional Chinese medicine ophthalmologists. The team members collaborate closely in various aspects such as lesson preparation, teaching, and case discussion. The Western medicine teachers are responsible for explaining the modern diagnostic and treatment standards of the disease and the key points of surgery, while the traditional Chinese medicine teachers focus on syndrome differentiation and the selection of treatment methods and prescriptions.

### 2.4 Data collection instruments and indicators

#### 2.4.1 Questionnaire survey

The questionnaire was reviewed by two ophthalmology teaching experts (Content Validity Index, CVI = 0.92). Before the official distribution of the questionnaire, we conducted a pre-test and tested its Cronbach’s *α* coefficient, which was *α* = 0.86, indicating that the questionnaire had high reliability and stability. The questionnaire was designed using a five-point Likert scale (1 = very dissatisfied/strongly disagree to 5 = very satisfied/strongly agree) to assess students’ subjective perception, satisfaction, and changes in learning motivation toward the new teaching model. The questionnaire was distributed within one week after the intervention and collected on the spot.

The main items were categorized into three dimensions: learning engagement, autonomous learning ability, and comprehensive clinical skills. In learning engagement dimension, it included satisfaction with course content, active participation enthusiasm in CBL/PBL, and interest in integrated TCM-WM knowledge. In autonomous learning ability dimension, it included degree of assistance provided by the teaching model, improvement effect on autonomous learning ability, and ability to solve new problems independently. In comprehensive clinical skills dimension, it included improvement effect on case handling ability, confidence in developing TCM-WM combined treatment plans, and effectiveness in connecting TCM syndrome differentiation and Western pathology (Table 1).

**Table 1:** Structure and scale of new ophthalmology teaching model evaluation questionnaire.

Dimension Name	Specific Items	Likert Scale Description
Learning engagement	1. Satisfaction with course content 2. Active participation enthusiasm in CBL/PBL 3. Interest in integrated TCM-WM knowledge	5-point scale (1 = very dissatisfied/strongly disagree; 5 = very satisfied/strongly agree)
Autonomous learning ability	1. Degree of assistance provided by the teaching model 2. Improvement effect on autonomous learning ability 3. Ability to solve new problems independently	5-point scale (1 = very dissatisfied/strongly disagree; 5 = very satisfied/strongly agree)
Comprehensive clinical skills	1. Improvement effect on case handling ability 2. Confidence in developing TCM-WM combined treatment plans 3. Effectiveness in connecting TCM syndrome differentiation and Western pathology	5-point scale (1 = very dissatisfied/strongly disagree; 5 = very satisfied/strongly agree)

#### 2.4.2 Objective assessment indicators

We assess teaching effectiveness from three aspects, including knowledge mastery, behavioral indicators, and practical skill indicators.

The mastery of knowledge is assessed using a comprehensive examination score (on a 100-point scale), with the composition of the comprehensive score being 40% theoretical assessment, 30% virtual practical operation, and 30% case presentation. The results are compared before and after intervention.

Behavior indicators include classroom participation and frequency of independent literature review. Classroom participation is observed and recorded by two teachers, including students’ raising hands to ask questions and group discussions, and the average value is taken. The frequency of independent literature review is self-assessed and recorded by students, summarized weekly, and the average value is taken.

Practical skill indicators include Western medicine examination operations and traditional Chinese medicine characteristic operations. The Western medicine examination mainly assesses the standardization and accuracy of students’ intraocular pressure measurement, slit lamp operation, etc., and is independently scored by two teachers. The average value is taken and recorded on a percentage scale. The characteristic operation of traditional Chinese medicine mainly assesses the location of acupuncture and moxibustion points for students. Select 10 commonly used acupoints in ophthalmology, including Jingming, Cunzhu, and Sizhukong, and record the accuracy of acupoint positioning (correct cases/total cases * 100%).

### 2.5 Data quality control

The practical skills assessment adopts “double-blind scoring,” and the scoring teacher does not know the students’ pre intervention scores. Data entry is done by two people on a dual track basis, and statistical analysis is conducted after verifying that there are no errors.

### 2.6 Statistical analysis

All data were analyzed using SPSS version 25.0 (IBM Corp., Armonk, NY, USA). The Shapiro–Wilk test was first used to assess the normality of continuous variables. Normally distributed continuous data were expressed as mean ± standard deviation (x̄±s), and pre- and post-intervention values were compared using paired t-tests. Non-normally distributed continuous data were expressed as median and interquartile range [M (P25, P75)], and pre- and post-intervention comparisons were performed using the Wilcoxon signed-rank test. Categorical variables were presented as frequencies and percentages [n (%)], and pre- and post-intervention comparisons were conducted using the chi-square test; when the expected cell count was less than 5, the continuity-corrected chi-square test or Fisher’s exact test was applied. All statistical tests were two-sided, and a *p* value <0.05 was considered statistically significant.

## 3 Results

### 3.1 Student satisfaction with the new teaching model

156 students completed a satisfaction questionnaire, and the results showed that students were highly satisfied with various aspects of the new teaching mode, with an average score of over 4.4 points. The overall average satisfaction score was 4.54 ± 0.33 points, and the satisfaction and very satisfaction rates exceeded 94.0%. The “Improvement of case handling ability” project scored the highest (4.62 ± 0.30 points), while the “Satisfaction with equipment and training environment” project scored the lowest (4.45 ± 0.41 points), but still at a relatively high level. The average score for the “Learning engagement dimension” was 4.55 ± 0.32 points, for the “Autonomous learning ability dimension” was 4.51 ± 0.35 points, and for the “comprehensive clinical skills dimension” was 4.59 ± 0.29 points, all indicating high levels of satisfaction across the key competencies (Table 2).

**Table 2:** Student satisfaction with the new teaching model.

Project	Average score (x ± s) (5-point scale)	Satisfied/very satisfied ratio (%)
Learning engagement dimension (overall)	4.55 ± 0.32	97.10
Satisfaction with course content	4.48 ± 0.35	98.25
Active participation in CBL	4.56 ± 0.31	97.33
Interest in integrated TCM-WM knowledge	4.61 ± 0.30	95.73
Autonomous learning ability dimension (overall)	4.51 ± 0.35	95.57
Improvement effect of PBL self-learning ability	4.54 ± 0.33	94.76
The degree of assistance of the new teaching model	4.59 ± 0.28	96.29
Ability to solve new problems independently	4.40 ± 0.44	95.67
Comprehensive clinical skills (overall)	4.59 ± 0.29	96.79
Improvement effect of case handling ability	4.62 ± 0.30	94.80
Confidence in developing TCM-WM combined treatment plans	4.55 ± 0.27	98.05
Effectiveness in connecting TCM syndrome differentiation and Western pathology	4.60 ± 0.31	97.53
Satisfaction with equipment and training environment	4.45 ± 0.41	96.33
Overall average satisfaction	4.54 ± 0.33	96.00

### 3.2 Effects on learning engagement and knowledge mastery

Under the old teaching mode, the active participation rate of students in the classroom was 64.0 ± 8.5%, while under the new teaching mode, it was 92.0 ± 4.5%. There was a significant statistical difference between the two (*t* = 9.50, *p* < 0.001). Under the old teaching mode, the average score of students’ comprehensive exams was 77.0 ± 5.5 points, while under the new teaching mode, the average score was 92.0 ± 4.0 points. There was a significant difference between the two (*t* = 8.52, *p* < 0.001). Under the traditional teaching mode, the frequency of students’ independent literature review is 1.2 ± 0.3/week. Under the new teaching mode, the frequency has increased to 3.5 ± 0.4/week, and the difference between the two is also statistically significant (*t* = 10.21, *p* < 0.001). These results indicate that the new teaching model significantly improves students’ classroom participation, knowledge mastery, and learning initiative (Table 3).

**Table 3:** Comparison of student learning indicators before and after the new teaching mode.

Observation indicators	Old teaching mode	New teaching mode	t/χ^2^	*P* value
Active classroom participation rate (%)	64.0 ± 8.5	92.0 ± 4.5	9.50	<0.001
Frequency of independent literature review (times/week)	1.2 ± 0.3	3.5 ± 0.4	10.21	<0.001
Comprehensive average score of the exam (points)	77.0 ± 5.5	92.0 ± 4.0	8.52	<0.001
Accuracy of acupuncture and moxibustion point location (%)	84.0	95.0	12.15	<0.001
Average score of Western medical examination operation (points)	80.5 ± 4.2	95.5 ± 3.1	7.80	<0.001

### 3.3 Effects on practical skills

In terms of practical skills, the reform effect was also very significant. Under the old teaching mode, the accuracy rate of students’ acupuncture point localization was 84.0%, while under the new teaching mode, the accuracy rate significantly improved to 95.0% (*χ*^2^=12.15, *p* < 0.001). In the old teaching mode, the average score of students in the Western medicine examination item slit lamp examination was 80.5 ± 4.2, while in the new teaching mode, the average score was 95.5 ± 3.1 (*t* = 7.80, *p* < 0.001). The difference between the two was significant (*t* = 7.80, *p* < 0.001). These results indicated that the new teaching model significantly improves students’ practical skills (Table 3).

## 4 Discussion

The combination of traditional Chinese and Western medicine has its unique clinical value in the field of modern medicine (15, 16). In ophthalmic treatment, the combination of traditional Chinese and Western medicine also has advantages. For example, in the case of clinically challenging optic neuropathy, a single treatment method often has limited effectiveness. The collaborative diagnosis and treatment of traditional Chinese and Western medicine provides new hope for such patients (17). Zhu et al. (18) found that, Tang Luo Ning, a traditional Chinese compound prescription, can treat diabetic peripheral neuropathy by improving mitochondrial dynamics. In the treatment of dry eye syndrome, Compound herbs, including Chi-Ju-Di-Huang-Wan and Qiming granule, can effectively alleviate dry eye symptoms (19). Moreover, patients with dry eye disease who were treated with Western medicine combined with TCM experienced significantly magnified therapeutic effects and reasonable costs of treatment (20). The integrated model of traditional Chinese medicine and Western medicine combines the overall regulation of traditional Chinese medicine with the precise targeted treatment of Western medicine, greatly enriching and expanding the treatment methods for ophthalmic diseases (21, 22).

Previous studies have found that students generally feel confused about how to “integrate” traditional Chinese and Western medicine (23). Under the traditional training model, students are often required to switch back and forth between two completely different discursive systems (24): when studying anatomy and physiology, they adopt a Western medical mode of thinking oriented toward reductionism, whereas when learning about zang–fu organs and meridians, they must shift to a traditional Chinese medicine mindset that takes holism as its core (25, 26). Over time, this easily leads to a clear sense of cognitive fragmentation. On the one hand, students can fluently recite pathological mechanisms in Western medicine and accurately reproduce syndrome classifications in traditional Chinese medicine; on the other hand, these two bodies of knowledge are difficult to connect in real clinical settings. When confronted with complex clinical problems, they are forced to oscillate between the two frameworks, making it hard to achieve genuine coherence and integration (27). The core value of the three-dimensional teaching model lies in fundamentally reconstructing the connections between different domains of knowledge. Its goal is no longer for students to learn the two medical systems separately, but to use both systems to understand the same disease. Instead of relying on superficial integration strategies such as “learning traditional Chinese medicine first and then Western medicine” or simply juxtaposing the two for comparison, this model takes the disease or core pathogenesis as the central axis and deliberately guides students (28), for the same clinical problem, to engage simultaneously with Western medicine’s “microscopic pathology” and traditional Chinese medicine’s “macroscopic syndromes.”

From a design perspective, the three-dimensional ITCWM teaching model was deliberately constructed around three core elements. First, a disease-centred, dual-track reasoning framework was adopted, in which each clinical case is analyzed simultaneously from the perspectives of Western disease diagnosis and TCM syndrome differentiation. Second, CBL and PBL are integrated to create authentic clinical scenarios, guiding students to shift from passive reception of knowledge to active, problem-driven inquiry. Third, a dedicated ITCWM training centre aligns classroom teaching with hands-on practice by providing matched equipment and simulation environments. Through this alignment of teaching objectives, content, methods, and assessment, the model aims to cultivate students’ ability to apply integrated TCM–WM thinking in real clinical decision-making rather than merely memorising two parallel theoretical systems.

We have developed a three-dimensional teaching method of “Western Medicine Diagnosis Traditional Chinese Medicine Syndrome Differentiation Collaborative Intervention,” successfully overcoming the drawbacks of the disconnect between traditional Chinese and Western medicine theories in teaching. This approach can encourage students to follow a logical and rigorous dual line thinking path when dealing with cases, organically combining the precise pathological analysis of Western medicine with the holistic concept and individualized differentiation of traditional Chinese medicine. Our research found that the use of three-dimensional teaching methods significantly improved students’ comprehensive exam scores, indicating a significant improvement in their ability to integrate traditional Chinese and Western medicine knowledge. This result is consistent with previous research on integrated traditional Chinese and Western medicine teaching (29, 30).

Many TCM featured operations, such as acupuncture and moxibustion, massage, cupping, etc., have unique functions and auxiliary effects in the treatment of many diseases (31–34). The inheritance of traditional Chinese medicine characteristic technologies relies on sufficient training resources and standardized assessment standards (35). Some teaching institutions have insufficient investment in traditional Chinese medicine teaching staff, training venues, and equipment, resulting in the marginalization of traditional Chinese medicine characteristic operation training in daily teaching, and students’ lack of traditional Chinese medicine operation ability (36). We have established an ophthalmic operation center of integrated traditional Chinese and western medicine, providing acupuncture and moxibustion simulators, traditional Chinese medicine fumigants and other special equipment, providing necessary hardware support for students. At the same time, we have included acupuncture and moxibustion point positioning into the compulsory examination indicators, which has significantly improved the students’ practical ability. After the operation training, the accuracy rate of acupuncture and moxibustion point positioning of students was significantly improved, indicating that the new model effectively made up for the shortcomings of traditional Chinese medicine practice teaching. It is worth noting that the teaching of traditional Chinese medicine characteristic techniques needs to be combined with modern technology, such as displaying the anatomical relationship between eye acupoints, nerves, and blood vessels through augmented reality (AR) systems, which can further enhance students’ understanding of acupoint positioning accuracy (37), and this is also one of the directions for future teaching optimization.

PBL and CBL aim to create authentic medical scenarios and encourage students to actively shift their mindset from “what I have” to “what I can do,” and from the “being taught” model to the “I want to learn” paradigm (38, 39). Establishing a group learning mode through PBL allows for deeper communication between teachers and students, thus achieving personalized teaching objectives (40, 41). CBL prepares clinical case materials and guides teachers to help students develop more effective clinical comprehensive thinking habits (42). This study combines PBL and CBL teaching methods, complementing and promoting each other. We found that under the new model, students’ classroom participation and frequency of independent literature review have significantly increased, which strongly confirms the advantages of PBL and CBL in stimulating students’ interest and initiative in learning. In addition to objective performance indicators, the questionnaire results further confirmed the acceptability and perceived value of the new model. The overall satisfaction score reached 4.54 ± 0.33 on a five-point scale, and more than 96% of students reported being satisfied or very satisfied with the course. Across the three predefined dimensions, the mean scores for learning engagement, autonomous learning ability, and comprehensive clinical skills were all above 4.5, indicating that students clearly recognized the model’s ability to stimulate participation, support self-directed learning, and enhance integrated clinical competence. These subjective outcomes are consistent with the observed improvements in examination scores, literature-review frequency, and practical skills, suggesting that the design logic of the three-dimensional ITCWM curriculum was well aligned with students’ learning needs and expectations. This result is consistent with previous research findings (43–45).

We need to know that during the clinical rotation process of these medical students, the ophthalmology course only had 32 credit hours, and the total teaching time was very limited. Given the depth and breadth of both TCM and WM content, the goal of this model in such a short time frame was not to cultivate clinical doctors with independent practice ability, but to focus on cultivating students’ clinical comprehensive thinking ability and basic clinical ability. The research results found that under the new mode, students’ autonomous learning ability and learning participation have been significantly improved, indicating that the new mode successfully stimulated students to engage in active self-learning and continuous learning outside the scheduled class time, effectively extending the learning process to more than 32 h. This foundation in integrated clinical thinking and self-directed learning capability is the true measure of success for this clerkship intervention, providing them with the essential tools needed for future independent clinical practice and residency training. Therefore, the observed significant improvements in exam scores and skill mastery are interpreted as the successful attainment of these foundational, integrated competencies, rather than the achievement of comprehensive, advanced diagnostic and therapeutic mastery.

## 5 Conclusion

In summary, this study enhanced the quality of clinical ophthalmology teaching by constructing and implementing a three-dimensional integrated teaching model that strengthens practical training, improves faculty development, and refines the teaching evaluation system. The new model helped to address key shortcomings of traditional teaching and significantly improved the core competencies and overall competitiveness of medical students. It effectively stimulated students’ interest in integrating knowledge from traditional Chinese medicine and Western medicine, leading to a marked increase in active classroom participation. By integrating CBL and PBL, the model increased students’ frequency of independent literature searching and strengthened their ability to independently analyze clinical scenarios and solve new problems. At the same time, the three-dimensional teaching model supported the development of a clear, dual-track clinical reasoning framework that links Western disease diagnosis with traditional Chinese medicine syndrome differentiation. Overall, the comprehensive evaluation suggests that this teaching model is effective and provides a scientifically and pedagogically sound approach for cultivating future ophthalmology professionals with the ability to integrate traditional Chinese and Western medicine.

## Data Availability

The original contributions presented in the study are included in the article/supplementary material, further inquiries can be directed to the corresponding author/s.
